# Response surface analysis of photocatalytic degradation of methyl tert-butyl ether by core/shell Fe_3_O_4_/ZnO nanoparticles

**DOI:** 10.1186/2052-336X-12-1

**Published:** 2014-01-06

**Authors:** Mojtaba Safari, Mohammad Hossein Rostami, Mehryana Alizadeh, Atefeh Alizadehbirjandi, Seyyed Ali Akbar Nakhli, Reza Aminzadeh

**Affiliations:** 1 Department Of Chemical Engineering, Amirkabir University Of Technology, Tehran, Iran; 2 Department Of Chemical And Petroleum Engineering, Sharif University Of Technology, Tehran, Iran

**Keywords:** Fe_3_O_4_/ZnO nanoparticles, Photocatalytic degradation, MTBE, Response surface modeling

## Abstract

The degradation of methyl tert-butyl ether (MTBE) was investigated in the aqueous solution of coated ZnO onto magnetite nanoparticale based on an advanced photocatalytic oxidation process. The photocatalysts were synthesized by coating of ZnO onto magnetite using precipitation method. The sample was characterized by X-ray diffraction (XRD), scanning electron microscopy (SEM), and vibration sample magnetometer (VSM). Besides, specific surface area was also determined by BET method. The four effective factors including pH of the reaction mixture, Fe_3_O_4_/ZnO magnetic nanoparticles concentration, initial MTBE concentration and molar ratio of [H_2_O_2_]/ [MTBE] were optimized using response surface modeling (RSM). Using the four-factor-three-level Box–Behnken design, 29 runs were designed considering the effective ranges of the influential factors. The optimized values for the operational parameters under the respective constraints were obtained at PH of 7.2, Fe_3_O_4_/ZnO concentration of 1.78 g/L, initial MTBE concentration of 89.14 mg/L and [H_2_O_2_]/ [MTBE] molar ratio of 2.33. Moreover, kinetics of MTBE degradation was determined under optimum condition. The study about core/shell magnetic nanoparticles (MNPs) recycling were also carried out and after about four times, the percentage of the photocatalytic degradation was about 70%.

**Electronic supplementary material:**

The online version of this article (doi:10.1186/2052-336X-12-1) contains supplementary material, which is available to authorized users.

## Introduction

Methyl tert-butyl ether (MTBE) is commercially used as an octane enhancer for gasoline. It can reach underground water resources in different ways such as leaking underground fuel tanks, leaking pipelines, tank overfilling, faulty construction at gas stations, spillage from vehicle accidents and home owner releases may result in contamination of ground and surface water resources[1, 2]. The admissible limit of MTBE in drinking water is 20–40 ppb[3, 4] which has resulted in the prevention of this material to be used as a gasoline additive since May 2006[5]. The toxicity of MTBE to animals and humans is well documented. It is well known that MTBE is carcinogenic to animals, which is due to diverse properties such as the existence of ether bond and long sub-branches (more than one carbon) in its structure. MTBE is known as a very resistant substance to natural degradation[6, 7]. In recent decades, many technologies have been devoted to MTBE degradation in water. Some of these technologies included adsorption on granular activated carbon (GAC), air stripping, advance oxidation processes (AOPs) and biodegradation[8]. Over the past three decades, AOPs were efficient methods for degradation of organic contaminants. An AOP is a photocatalysis process, which mineralizes and degrades the organic contaminants, accordingly[9]. Many researchers have studied the photocatalytic degradation of MTBE using TiO_2_ and metal-doped TiO_2_ in either powder or thin film form[9–11]. However, most of these studies have followed the classical method of optimizing one factor at a time (OFAT), which is a time consuming and laborious task. This method, also, does not consider the interactions among the operational factors. However, Response surface methodology (RSM) can combine mathematics and statistics to analyze the relative significance of various operating parameters even in complicated systems[12]. Hence, this method can be applied to determine the optimum conditions of various reactions in a more convenient way resulting in saving time, labor, and cost.

Many types of photocatalytic reactors have been proposed according to respective application demands; among them, however, a slurry type reactor has proved to be most attractive for degrading organic contaminants which dissolve in water namely in terms of reaction surface area per unit volume of the reactor[13]. Nonetheless, one of the main problems of the suspended photocatalyst system is that it requires a separation step to recover photocatalyst particles. In this case, a suitable technique such as centrifugation or filtration step is required to reuse fine photocatalyst particles[14].

In this work Fe_3_O_4_/ZnO core/shell composite catalyst was synthesized and then characterized by XRD, SEM and VSM. The magnetic core enhancing the separation properties of suspended particles from solution and the photocatalytic properties of the outer shell zinc oxide are used to destroy organic contaminants in wastewater[15]. The four effective parameters, optimized using RSM, were (i) pH, (ii) coating of ZnO onto magnetite concentration, (iii) MTBE initial, and (iv) molar ratio of [H_2_O_2_]_o_/[MTBE]_o._ In the end, kinetics of MTBE degradation was determined in optimum condition.

## Materials and methods

### Materials

Methyl tert-butyl ether (99.9%), ferric chloride (FeCl_3__6H_2_O), hydrogen peroxide (35% w/w), ferrous sulfate (FeSO_4__7H_2_O), zinc acetate (ZnAC_2__2H_2_O), aqueous ammonia (NH_3__H_2_O) HNO_3_ and NaOH were purchased from Merk. ammonium carbonate ((NH_4_)2CO_3_) purchased from Dae jung.

### Instruments

The instruments used for studying synthesized nanoparticles were XRD (Equinox 3000, Inel france), SEM (AIS2100, seron technology), BET (Autosorb-1, Quantachrome), 2 lamps (UVa 11W, Philips, Netherland), gas choromatography (GC) equipped with a helium ionization detector (HID) (Model GC-Acme 6100, Korea), vibration sample magnetometer (VSM, Meghnatis Daghigh Kavir Co., Iran), magnetic stirrer (Dalahan Labtech, LMS-1003) and digital pH meter (Elmetron, Cpc-501).

### Preparation of the photocatalyst

A co-precipitation method was used to synthesize the Fe_3_O_4_ magnetic nanoparticles (MNPs). Co-precipitation is a facile and convenient way to synthesize MNPs from aqueous salt solutions. This is accomplished by addition of ammonia to mixture of ferric chloride (0.5 M) and ferrous sulfate (0.5 M) with molar ratio of 1.75:1 under inert argon protection until pH value reached to 9. After 30 min stirring, the precipitate collected using a magnet and washed with deionized water until pH reached to 7. The modification process has been carried out via sonication of 4 g Fe_3_O_4_ and 200 ml sodium citrate (0.5 M) mixture for 20 min, which was then stirred for 12 h at 60°C under Ar protection. Afterwards, the precipitate collected and rinsed with acetone. The Fe_3_O_4_/ZnO core/shell MNPs were obtained by coating the modified Fe_3_O_4_ MNPs with direct precipitation using zinc acetate and ammonium carbonate. The modified Fe_3_O_4_ added to 100 ml of deionized water and sonicated for 20 min to make a stable ferrofluid. Then, 30 ml of this ferrofluid was added into a flask to form Fe_3_O_4_/ZnO composite. Two solutions were made by adding 12.16 g ZnAC_2__2H_2_O and 7.6 g (NH_4_)2CO_3_ respectively into 100 ml of deionized water, and then, these two solutions were dropped slowly into the flask. Then the precipitate was collected and washed with water, aqueous ammonia (pH 9) and ethanol. Thereafter, the precipitate was dried under vacuum in 12 h and calcined according to desired calcination temperature and time[16].

### Experimental set up

Photocatalytic degradation of MTBE was performed in a slurry batch reactor which was configured with a cylindrical glass with one liter in volume. In order to control the temperature of the reactions, the reactor was provided with a jacket for water circulation. Two lamps (11w, Philips, Netherland), which were immersed in the solution, were applied to supply the UV radiation in the reactor. The reactor was tightly sealed and in order to ensure well-mixing during irradiation, the nanoparticles were dispersed in the solution under magnetic stir. Besides, the air was injected into the reactor to supply the required amount of oxygen for the photocatalysis.

### Experimental design by RSM method

Initially, preliminary experiments by following single factor study method were performed in order to find the most effective experimental parameters and their ranges affecting the photocatalytic degradation of MTBE. The selected parameters were catalytic dose, initial concentration of MTBE, initial concentration of H_2_O_2_ and pH.

The four selected experimental parameters were optimized using RSM considering them as independent variables and removal percentages of MTBE as response variables. By applying Box-Behnken design experiments, the required number of experiments were designed. This method was used because it is very efficient and does not contain any point at the vertices of the cubic region formed by the upper and lower limits of the variables. Such design along with RSM is widely applied for optimization of various physical, chemical and biological processes[17, 18].

**Figure 1:**
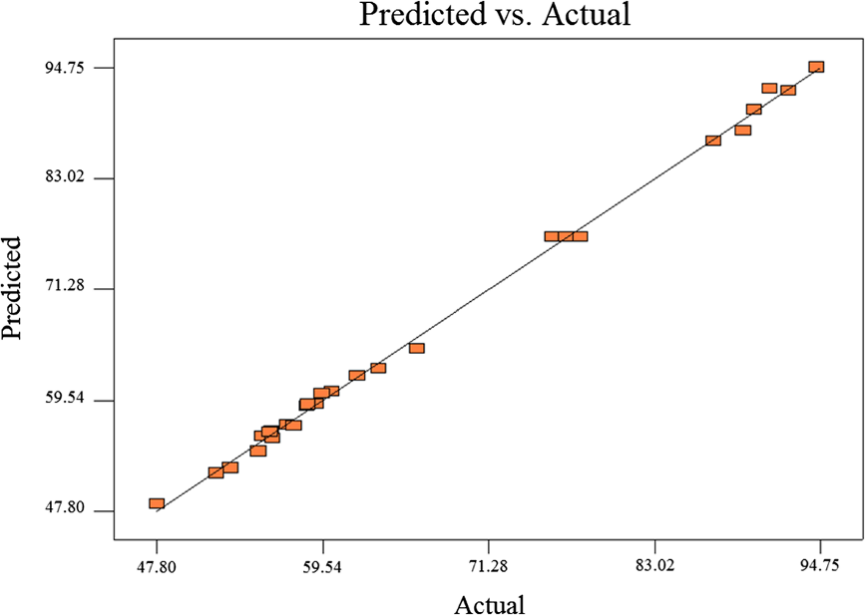
**Plot of the actual and predicted values for %MTBE removal.**

As expressed in equation 1, the results were fitted to an empirical quadratic polynomial model for the aforesaid parameters using RSM.

1 $$\begin{array}{l} Y=\beta_{0}+\beta_{1}A+\beta_{2}B+\beta_{3}C+\beta_{4}D+\beta_{11}A^{2}\\ \;+\beta_{22}B^{2}+\beta_{33}C^{2}+\beta_{44}D^{2}+\beta_{12}\text{AB}+\beta_{23}\text{BC}\\ \;+\beta_{31}\text{CA}+\beta_{41}\text{DA}+\beta_{42}\text{BD}+\beta_{34}\text{CD} \end{array}$$

where Y denotes the response variable, β_0_ the intercept, β_1_,β_2_,β_3_,β_4_ the coefficients of the independent variables, β_11_,β_22_,β_33_,β_44_ the quadratic coefficients, β_12_,β_23_,β_31_,β_41_,β_42_,β_34_ the interaction coefficients and A, B, C, D are the independent variables. Multivariate regression analysis and optimization process were performed by means of RSM and using Design Expert software (version 6.0.8, Stat Ease Inc., USA). The obtained values from analysis of variance (ANOVA) were found significant at p < 0.05. The optimum values for the independent variables were found using three-dimensional response surface analysis of the independent and dependent variables. The designed experiments plus the experimental and predicted values of the response are presented in Table 1. Also, the variations are shown in Figure 1.

**Table 1:** **Box–Behnken experiments along with actual and predicted values of responses**

Std	Run	B, initial MTBE concentration (ppm)	A, catalytic dose (g/L)	C, pH	D, initial H2O2 (ppm)	Y, COD MTBE removal (%)	
						Actual	Predicted
7	1	100	1	7	10	59.1	59.19
11	2	50	2.5	7	10	92.5	92.28
3	3	50	2.5	9	5	90.1	90.27
24	4	100	2.5	9	10	59.5	60.25
9	5	50	2.5	7	0	94.5	94.75
6	6	100	4	7	0	58.4	58.97
2	7	150	2.5	5	5	55.3	55.78
23	8	100	2.5	5	10	63.5	62.95
29	9	100	2.5	7	5	77.8	76.78
12	10	150	2.5	7	10	56	55.5
22	11	100	2.5	9	0	62	62.12
10	12	150	2.5	7	0	57	56.97
28	13	100	2.5	7	5	76.8	76.78
13	14	100	1	5	5	58.5	58.12
5	15	100	1	7	0	60.2	60.45
15	16	100	4	5	5	57.5	56.84
4	17	150	2.5	9	5	53	52.38
17	18	50	1	7	5	89.3	88.07
27	19	100	2.5	7	5	75.8	76.78
20	20	150	4	7	5	47.8	48.6
1	21	50	2.5	5	5	91.2	92.47
14	22	100	1	9	5	55.8	56.22
26	23	100	2.5	7	5	77.3	76.78
19	24	50	4	7	5	87.2	86.94
16	25	100	4	9	5	55	54.13
21	26	100	2.5	5	0	66.2	65.02
8	27	100	4	7	10	55.9	56.3
25	28	100	2.5	7	5	76.2	76.78
18	29	150	1	7	5	52	51.84

## Results and discussion

### Characterization of MNPs

**Figure 2:**
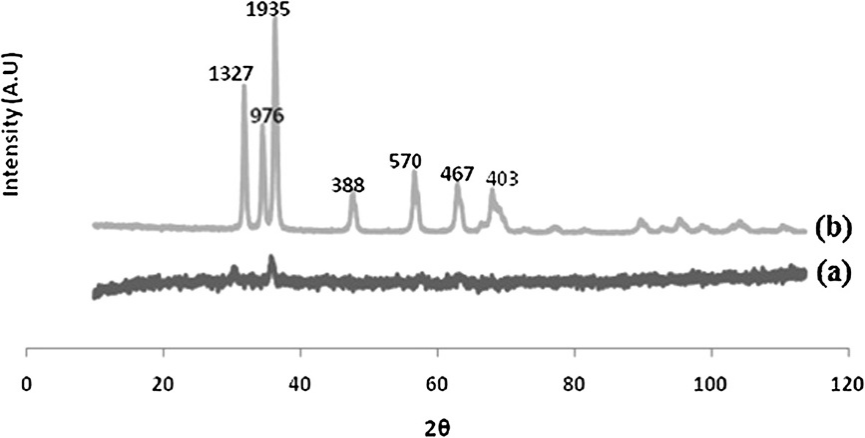
**XRD pattern of (a): modified Fe3O4 and (b): Fe3O4/ZnO core/shell.**

The X-ray diffraction pattern of modified Fe_3_O_4_ sample and Fe_3_O_4_/ZnO core/shell is presented in Figure 2. The average crystallite size was calculated using the Debye–Scherrer equation d= Kλ/(βcosθ) were about 13.9 nm (a), 11.2 nm (b) for modified Fe_3_O_4_ and Fe_3_O_4_, respectively. According to Figure 2b it is shown that after coating some enhances in peak intensity was caused by overlapping of Fe_3_O_4_ peaks.

**Figure 3:**
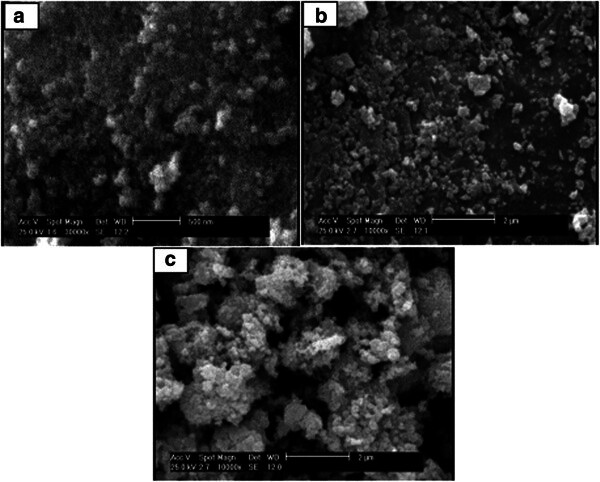
**SEM images of (a): Fe3O4 (b): modified Fe3O4 (c) Fe3O4/ZnO core/shell.**

In addition, Figure 3 represents SEM images of the samples. The SEM photographs of Fe_3_O_4_ MNPs before and after treating with sodium citrate are shown in Figure 3a and b respectively. It is shown that the dispersion of modified iron oxide is better than unmodified one. Figure 3c represents Fe_3_O_4_/ZnO core/shell particles which their average particle size was obtained about 60 nm.

**Figure 4:**
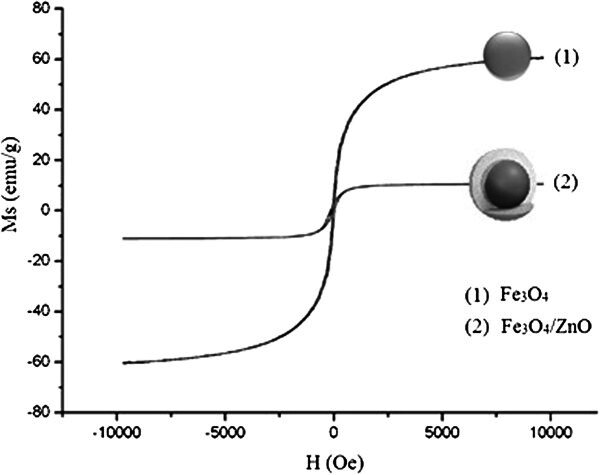
**Magnetic hysteresis curves of (1): modified Fe3O4 (2): Fe3O4/ZnO core/shell.**

The magnetic properties of MNPs are illustrated in Figure 4. It demonstrates that the coating process did not change the superparamagnetism of MNPs.

BET surface areas were determined using 3-points method for Fe3O4/ZnO nanoparticles which was 65 m^2^/gr.

### Statistical analysis

To acquire a desirable model, The results are summarized in a common ANOVA table. The ANOVA table for removal percentage of MTBE response is exhibited in Table 2. The R-square is found to be 0.99, which is close to 1, which implies that about 99% of changes in the data can be explained by the model. The lack-of-fit P value of 0.3855 shows that the lack-of-fit is not significant relative to net error. For a predictive model the value of Lack of Fit should be not significant.

**Table 2:** **ANOVA for response surface reduced quadratic model- analysis of variance**

Source	Sum of squares	Degrees of freedom	Mean square	F value	p-value prob > F	
Model	5867.65	14	419.12	487.39	<0.001	Significant
Residual	12.04	14	0.86			
Lack of fit	9.43	10	0.94	1.45	0.3855	Not significant
Pure error	2.61	4	0.65			
Cor total	5879.69	28		R-squared	0.9980	
				Adj R-squared	0.9959	
				Adeq precision	69.192	

Following the experimental design (Table 2), empirical second order polynomial equations are developed for the removal percentage of MTBE in terms of the three independent variables as is expressed in equation 2.

2 $$\begin{array}{l} \%\text{MTBE removal}=‒4.7400‒0.6688*\text{MTBE}+28.1254*\text{pH}\\ +26.8259*\text{TiO}2+2.1927*H2O2+0.0016*{\text{MTBE}}^{2}‒2.0423*pH^{2}\\ ‒5.3474*\text{TiO}2^{2}‒0.2408*H2O2^{2}‒0.0030*\text{MTBE}*\text{pH}+0.0010\\ {}*\text{MTBE}*H2O2+0.0167*\text{pH}*\text{TiO}2+0.0050*\text{pH}*H2O2\\ ‒0.0467*\text{TiO}2*H2O2 \end{array}$$

**Figure 5:**
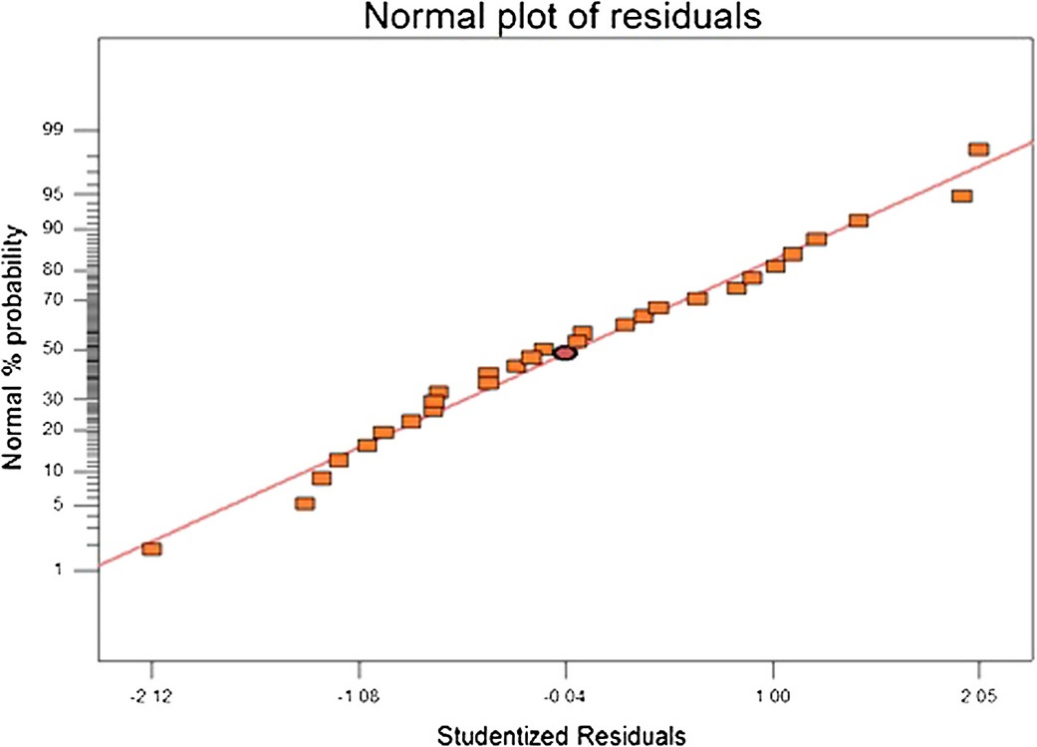
**Normal probability plot of residual for %MTBE removal.**

**Figure 6:**
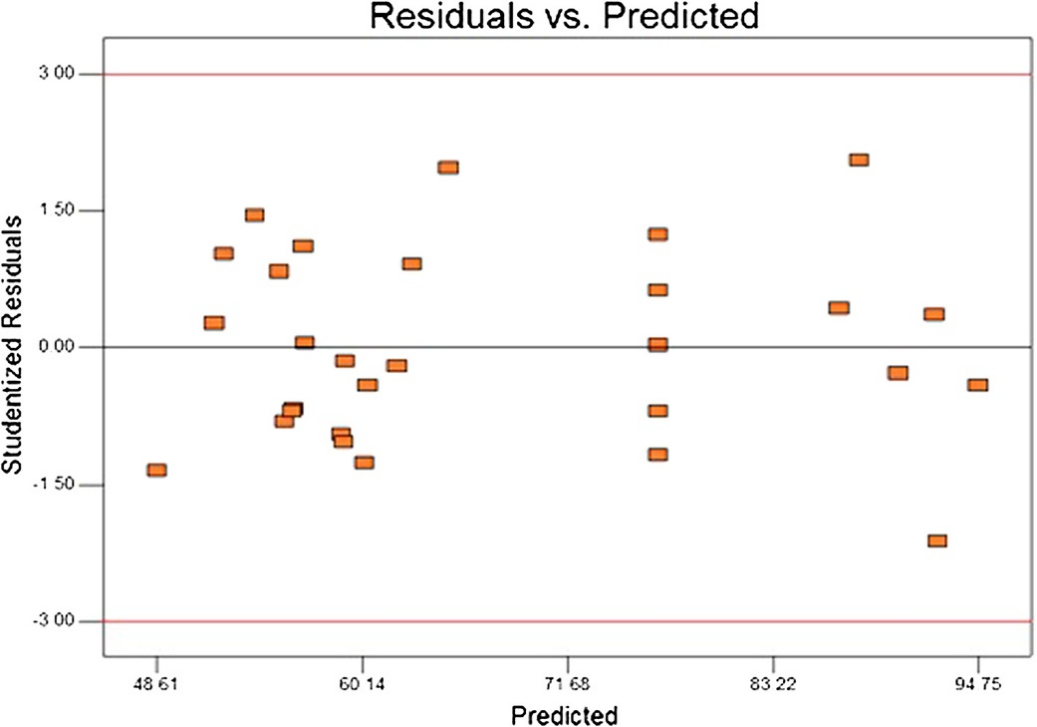
**Plot of residual vs. predicted response for %MTBE removal.**

**Figure 7:**
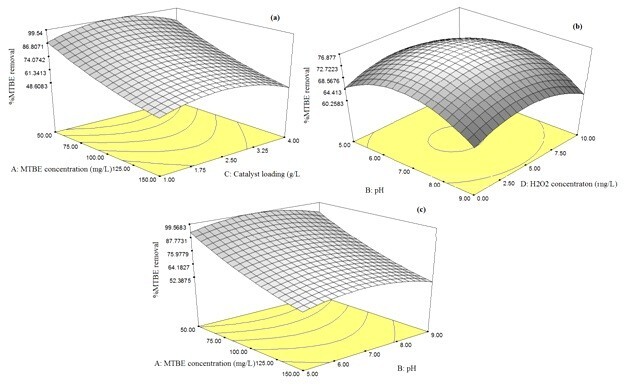
**Effect of catalyst loading, Initial MTBE concentration, H2O2 concentration and pH on %MTBE removal. (a)**: pH=7, H2O2 concentration= 5 mg/L; **(b)**: Initial MTBE concentration= 100 mg/L, Catalyst loading= 2.5 g/L; **(C)**: Catalyst loading= 2.5 g/L, H2O2 concentration= 5 mg/L.

The ANOVA of the second order quadratic polynomial model (F = 487.4, p < 0.0001) indicates that the model is significant, i.e. there is only a chance of 0.01% for occurrence of the model’s F-value due to the noise. The ANOVA regarding the regression models’s coefficient of the removal percentage of MTBE is presented in Table 3 as an extra tool to check the final model’s adequacy. The normal probability plot (scatter diagram) for the studentized residuals is illustrated in Figure 5. The points on this plot lie reasonably close to a straight line, confirming that the errors have a normal distribution with a zero mean and a constant. The curvature P-value < 0.0001 indicates that there is a significant curvature (as measured by the difference between the mean center points and the mean factorial points) in the design space. As a result, a linear model along with the interaction terms giving a twisted plane was not adequate to explain the response. Besides, plots of the residuals in Figure 6 reveal that they have no obvious pattern, and their structure is rather abnormal. Moreover, they indicate equal scatter above and below the x-axis, implying the proposed model’s adequacy, so there is no reason to suspect any violation. The optimum conditions for the maximum degradation of MTBE, that is selected with regard to proximity to the natural pH and using lowest catalyst loading, shown in Table 4 and the effect of the independents variable on the desirability shown in Figure 7.

**Table 3:** **ANOVA results for the coefficients of quadratic model for %MTBE removal**

Factor	Coefficient estimate	Degree of freedom	Standard error	95% confidence interval low	95% confidence interval high	F-value	p-value
Intercept	76.780	1	0.41	75.89	77.67	-	-
A-MTBE	-18.642	1	0.27	-19.22	-18.07	4849.47	< 0.0001
B-pH	-1.400	1	0.27	-1.97	-0.83	27.35	0.0001
C-Catalyst	-1.092	1	0.27	-1.67	-0.52	16.63	0.0011
D-H2O2	-0.983	1	0.27	-1.56	-0.41	13.49	0.0025
A2	4.118	1	0.36	3.34	4.90	127.94	< 0.0001
B2	-8.169	1	0.36	-8.95	-7.39	503.39	< 0.0001
C2	-12.032	1	0.36	-12.81	-11.25	1091.95	< 0.0001
D2	-6.019	1	0.36	-6.80	-5.24	273.29	< 0.0001
AB	-0.300	1	0.46	-1.29	0.69	0.42	0.5281
AC	-0.525	1	0.46	-1.52	0.47	1.28	0.2765
AD	0.250	1	0.46	-0.74	1.24	0.29	0.5982
BC	0.050	1	0.46	-0.94	1.04	0.01	0.9157
BD	0.050	1	0.46	-0.94	1.04	0.01	0.9157
CD	-0.350	1	0.46	-1.34	0.64	0.57	0.4628

**Table 4:** **The optimum conditions selected for the maximum possible the percentage of MTBE removal**

	Number	A, initial MTBE concentration (g/L)	B, catalytic dose (g/L)	C, pH	D, initial H2O2 (ppm)	MTBE removal (%)	Desirability	
Solutions	8	55.02	7.09	2.31	2.16	95.407	1	Selected

### Effect of Initial pH

pH is one of the most crucial parameters in photocatalytic degradation of organic contaminants. Figure 7b and c show the percent of degradation efficiency for several initial pH conditions. The degradation efficiency increases as the pH value is incremented from 5 to 7 and then adversely decreases with the increased value of pH from 7 to 9. The pH of the solution has complex effects on the photocatalytic oxidation reaction. However, in general, the pH effect depends on the type of pollutant and zero point charge (ZPC) of semiconductor (catalyst) in the oxidation process. Because, the pH of the solution affects the electrostatic force between the catalyst surface and the pollutant. Interactions among the semiconductor surface, solvent molecules, substrate and charged radicals formed during the reaction, the interpretation of the effect of pH on obtained results from photocatalytic degradation cannot be expressed as a whole and this phenomenon should be tested in the laboratory for each type of pollutant or should be found through available references at desired operating conditions[19].

The phenomenon can be explained in terms of the zero point charge location (isoelectric point) of the Fe_3_O_4_/ZnO. In acidic pH, MTBE will be protonized to carry the positive charge while the surface of Fe_3_O_4_/ZnO is electropositive. Therefore, the acidic pH does not favor the adsorption of MTBE on the Fe_3_O_4_/ZnO particles. When pH is alkaline, MTBE is neutral, but the surface of Fe_3_O_4_/ZnO is electronegative. Hence, adsorption of MTBE on the Fe_3_O_4_/ZnO particles in alkaline pH was less than that in neutral pH. According to results obtained from Figure 7b and c, natural pH was the best pH value for degradation of MTBE in this study[10].

### Effect of Fe_3_O_4_/ZnO MNPs concentration

**Figure 8:**
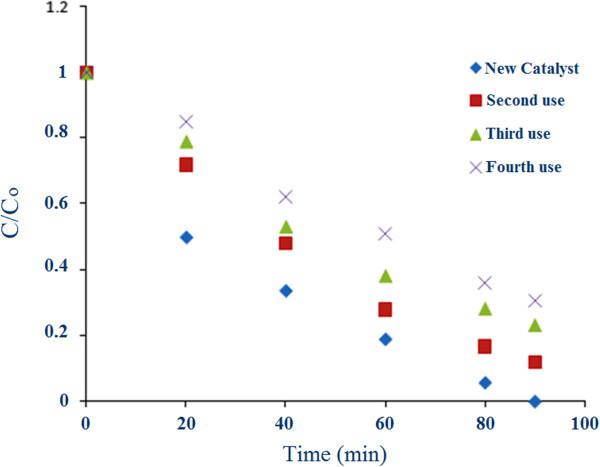
**MTBE concentration variation with time (at optimum condition).**

The percentage of degradation efficiency against catalyst loading is shown in Figure 7a for several initial Fe_3_O_4_/ZnO nanoparticles’ concentrations. The percentage of degradation efficiency increases along the increase in the catalyst loading from 1 to 2.5 g/L. However, by an increase in excess of 2.5 g/L, this percentage declines. It should be noted that, these results are highly contingent on the maintained experimental condition. As the amount of catalyst increases, the number of adsorbed photons and molecules increases as well due to an increase in the number of Fe_3_O_4_/ZnO nanoparticles. As a consequence, the particle density within the illumination area increases. This behavior can be attributed to the increase in opacity, which gives rise to a reduction in the radiation passage through the reactor[20]. It may also lead to Fe_3_O_4_/ZnO aggregation, reducing the active points on its surface to adsorb organic compounds and UV, thereby reducing the quantity of e-h+ and OH free radicals and affecting the degradation, accordingly[21]. After reusing of magnetic particles, a small decrease in the photocatalytic activity observed. After 4 times, the removal percentage decreased to about 70 percent. This decrease can be due to fouling of light-insensitive materials on active pores or loss of particles (Figure 8).

### Effect of initial MTBE concentration

Increase of initial MTBE concentration reduces its degradation as shown in Figure 7a and c. Similar results have been reported on the photocatalytic oxidation of other organic compounds interface[6–21]. At low MTBE concentrations, a larger number of water molecules will be adsorbed onto the available Fe_3_O_4_/ZnO nanoparticles, producing hydroxyl radicals and leading to a rapid oxidation process. On the other hand, at high MTBE concentration, there is a smaller portion of water molecules to free active sites, since the number of active sites remains the same. Consequently, the competition between the MTBE concentration and water molecules on adsorption increases and leading to a decline in the degradation rate.

### Kinetics of MTBE degradation

The Langmuir-Hinshelwood rate expression has been used to describe the relationship between the heterogeneous photocatalytic degradation rate and the initial pollutant concentration[22].

Experimental results in optimum condition (Figure 8) Indicated that the photodegradation rate of MTBE with UV/Fe_3_O_4_/ZnO/H_2_O_2_ fitted the Langmuir-Hinshelwood (L-H) kinetics model as follows:

3 $$-\frac{\text{dC}}{\text{dt}}=\frac{K_{r}K_{e}C}{1+K_{e}C}$$

It is assumed that the photodegradation of MTBE follows a first-order reaction; therefore the above equation can be simplified to an apparent first-order equation:

4 $$-\frac{\text{dC}}{\text{dt}}=\frac{K_{r}K_{e}C}{1+K_{e}C}=\text{KC}$$

where K_r_ is the reaction rate constant (mg/l.min), K_e_ is the adsorption coefficient of the MTBE (l/mg) and K_app_ is the apparent pseudo-first-order constant that is the multiplication product of the adsorption constant and the reaction constant. In this study, a reasonable agreement (R^2^ = 0.96) was obtained between the experimental results and the linear form of the L-H expression. Furthermore, this expression used values of 0.033(1/min) for K_app_.

### Effect of hydroxyl peroxide addition

Electron–hole recombination is the main energy-wasting step in the photocatalytic reaction. The prevention of this recombination is achieved by adding a proper electron donor or an acceptor to the system. Usually, molecular oxygen and hydrogen peroxide are used as electron acceptors in heterogeneous photocatalyzed reactions[23]. H_2_O_2_ can generate hydroxyl radicals through two ways as follows:

5 $$H_{2}O_{2}+\text{hv}\to 2\text{OH}{}^{\circ}$$

6 $$H_{2}O_{2}+e\to \text{OH}{}^{\circ}+OH^{‒}$$

The results are presented in Figure 7b show that the degradation rate had a maximum of the [H_2_O_2_]/[MTBE] molar ratio of 5. However, higher concentration of H_2_O_2_ can have a negative effect. This may be due to the formation of HO_2_^o^, a species that is significantly less reactive than HO^o^[24]. As shown in equations 7 and 8, the excess H_2_O_2_ molecules on the catalyst surface may also act as powerful scavengers of radicals[25, 26].

7 $${\text{OH}}^{o}+H_{2}O_{2}\to H_{2}O+{\text{HO}}_{2}^{o}$$

8 $$2{\text{HO}}_{2}^{o}\to H_{2}O_{2}+O_{2}^{o}$$

## Conclusions

In this study, Fe_3_O_4_/ZnO nanoparticles were successfully synthesized with average crystal size of 11.2 mm by precipitation method. Synthesized nanoparticles then utilized as a catalyst for the photocatalytic degradation of MTBE. The optimum levels of the operational parameters under the related constraint conditions were found at pH of 7.02, Fe_3_O_4_/ZnO MNPs concentration of 1.78 g/L, initial MTBE concentration of 89.14 mg/L, and [H_2_O_2_]/[MTBE] molar ratio of 2.33. In addition, according to the Langmuir-Hinshelwood kinetic model, the apparent pseudo-first-order constant was 0.033 (1/min) for experimental results under optimum conditions. Also the recycling and reuse of MNPs was significantly successful.

## Authors’ original submitted files for images

Below are the links to the authors’ original submitted files for images. Authors’ original file for figure 1 Authors’ original file for figure 2 Authors’ original file for figure 3 Authors’ original file for figure 4 Authors’ original file for figure 5 Authors’ original file for figure 6 Authors’ original file for figure 7 Authors’ original file for figure 8

## Abbreviations

XRD: X-ray diffraction
SEM: Scanning electron microscopy
VSM: Vibration sample magnetometer
RSM: Response surface modeling
MNP’s: Magnetic nanoparticles
GAC: Granular activated carbon
OFAT: Optimizing one factor at a time
GC: Gas chromatography
HID: Helium ionization detector
AVOVA: Analysis of variance
ZPC: Zero point charge
AOP’s: Advanced oxidation processes.
