# Supplementary figures and images for: Response surface analysis of photocatalytic degradation of methyl tert-butyl ether by core/shell Fe3O4/ZnO nanoparticles
Source: J Environ Health Sci Eng. 2014 Jan 6;12:1. doi: 10.1186/2052-336X-12-1 (PMC3937170; doi:10.1186/2052-336X-12-1)

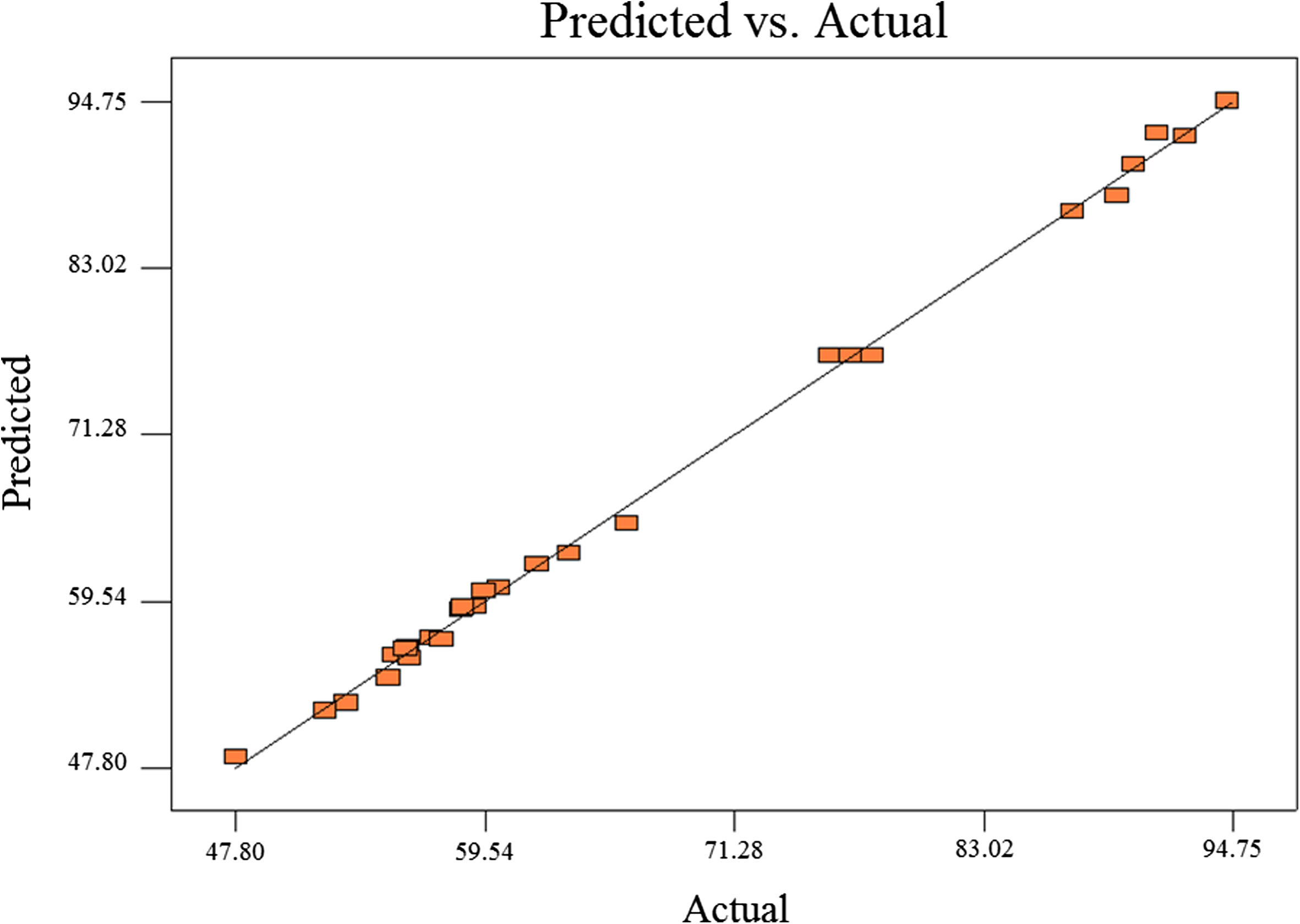

Supplement: Supplementary file 1 — Authors’ original file for figure 1 [file 40201_2012_5149_MOESM1_ESM.tiff]

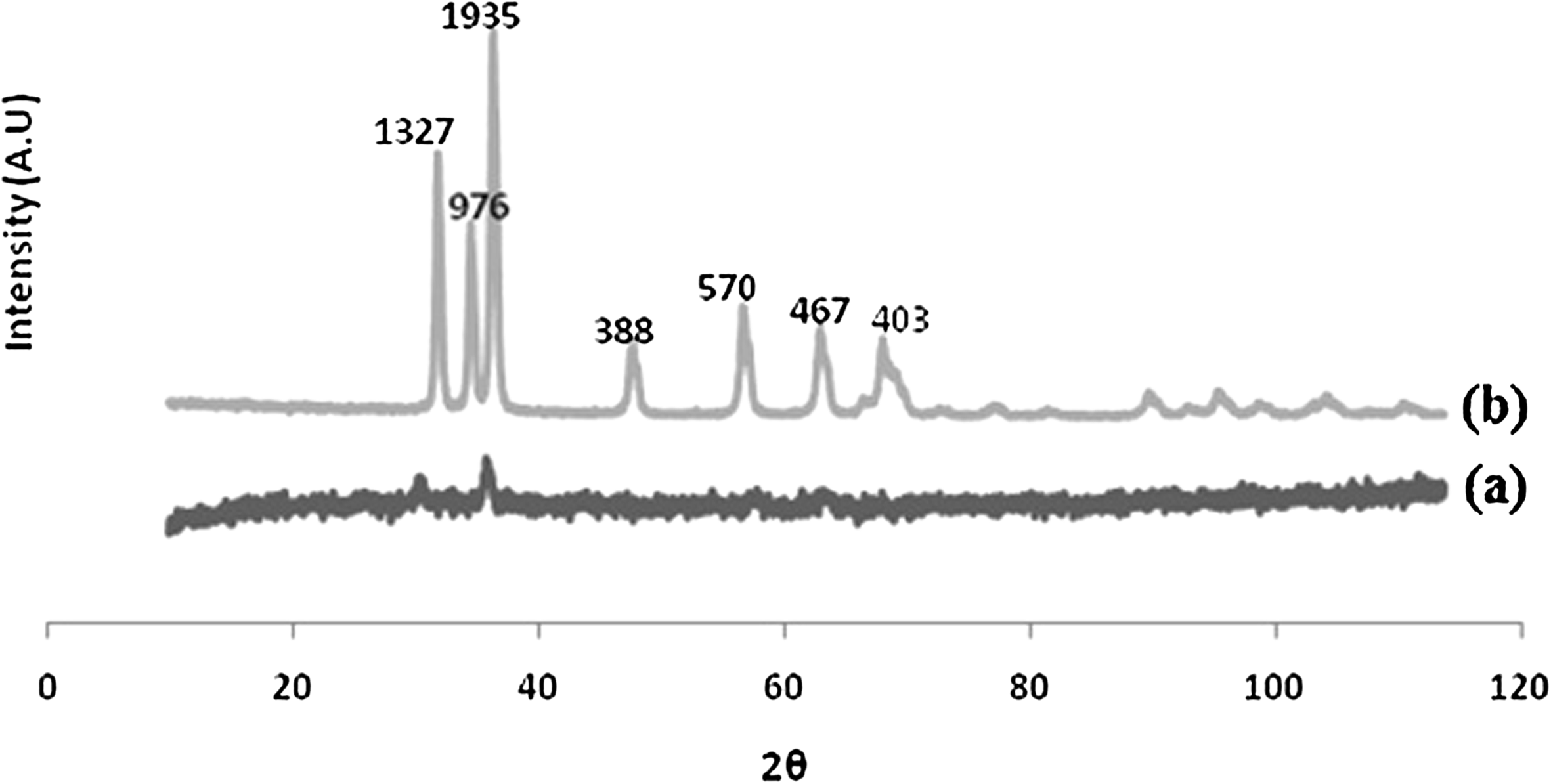

Supplement: Supplementary file 2 — Authors’ original file for figure 2 [file 40201_2012_5149_MOESM2_ESM.tif]

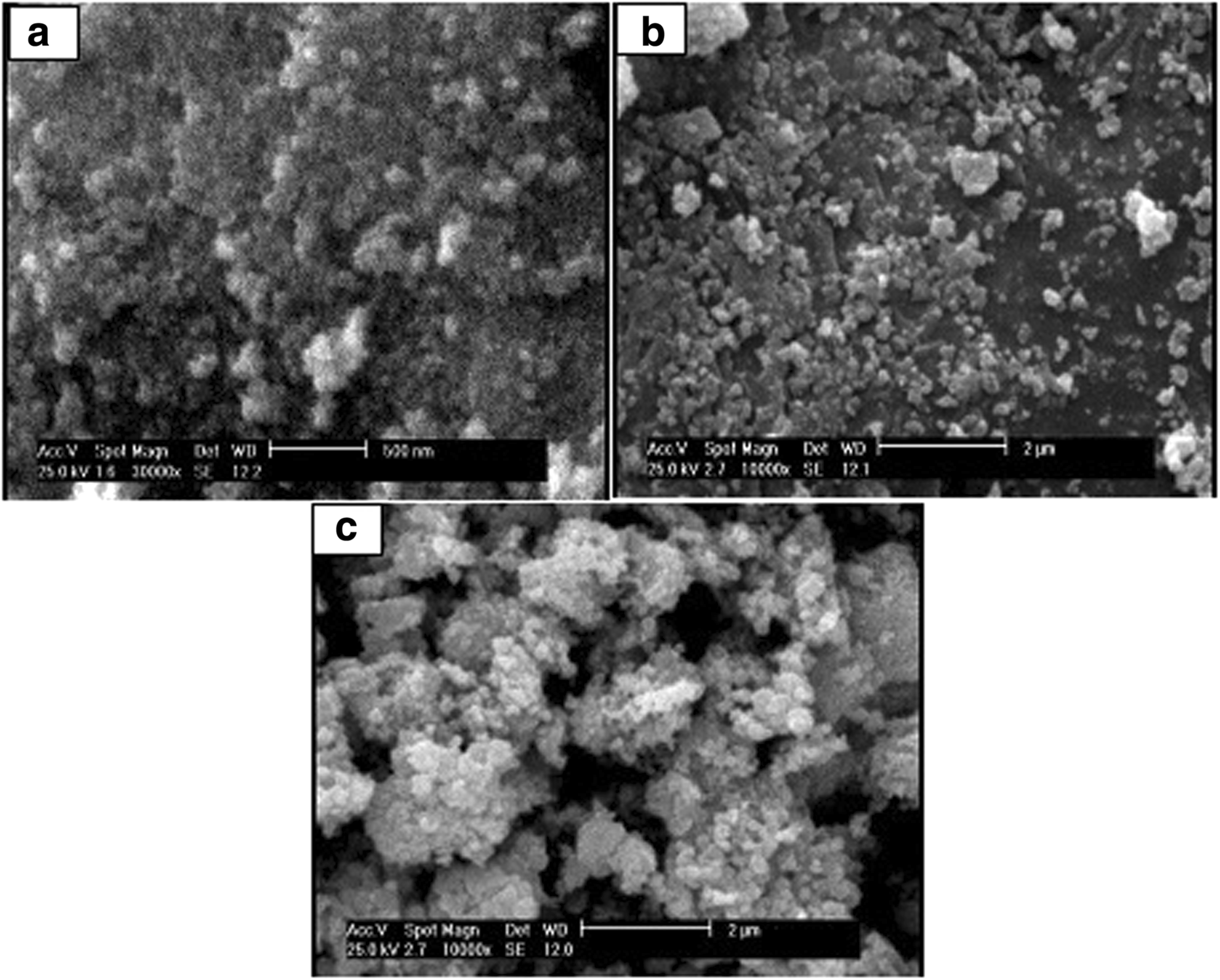

Supplement: Supplementary file 3 — Authors’ original file for figure 3 [file 40201_2012_5149_MOESM3_ESM.tif]

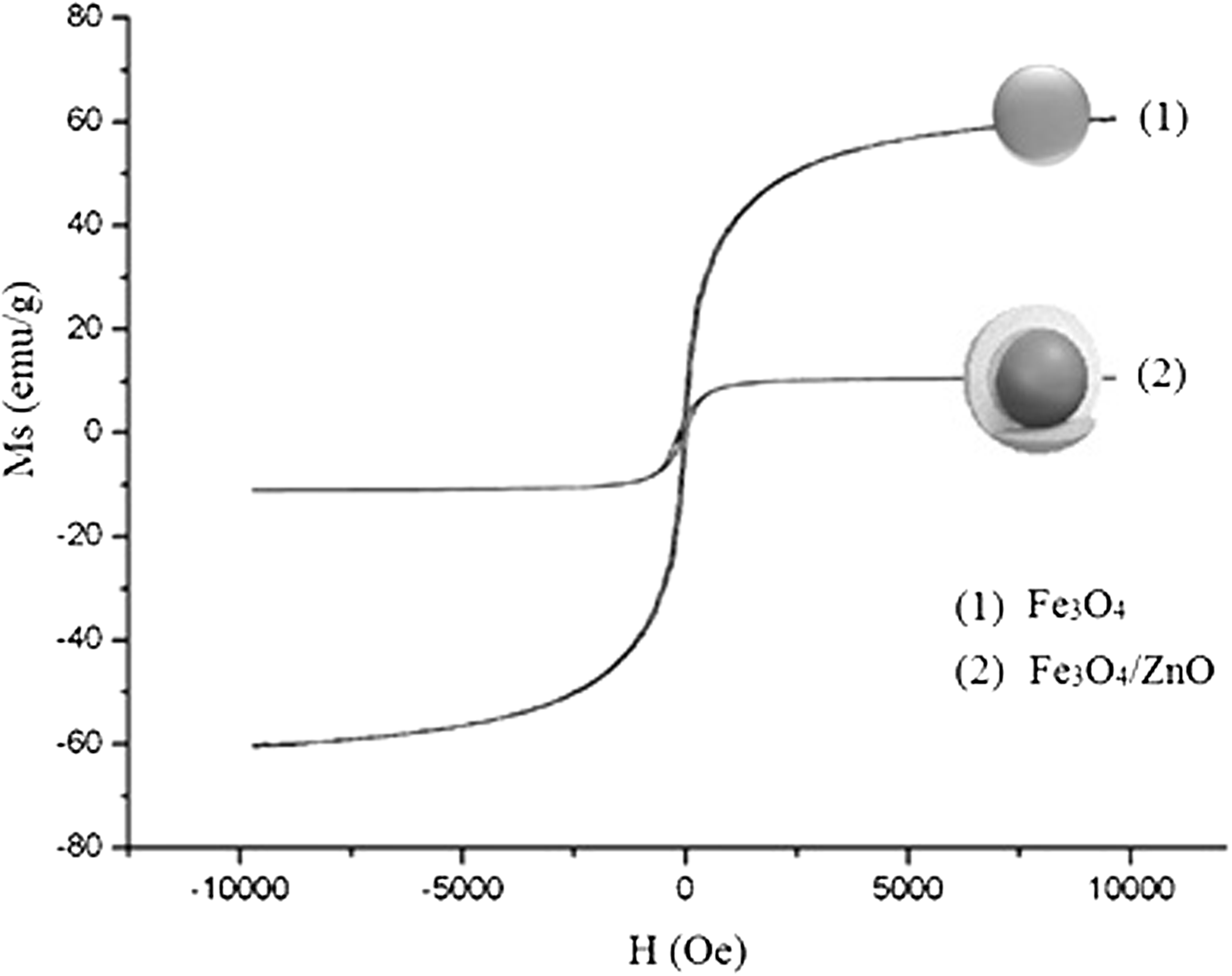

Supplement: Supplementary file 4 — Authors’ original file for figure 4 [file 40201_2012_5149_MOESM4_ESM.tif]

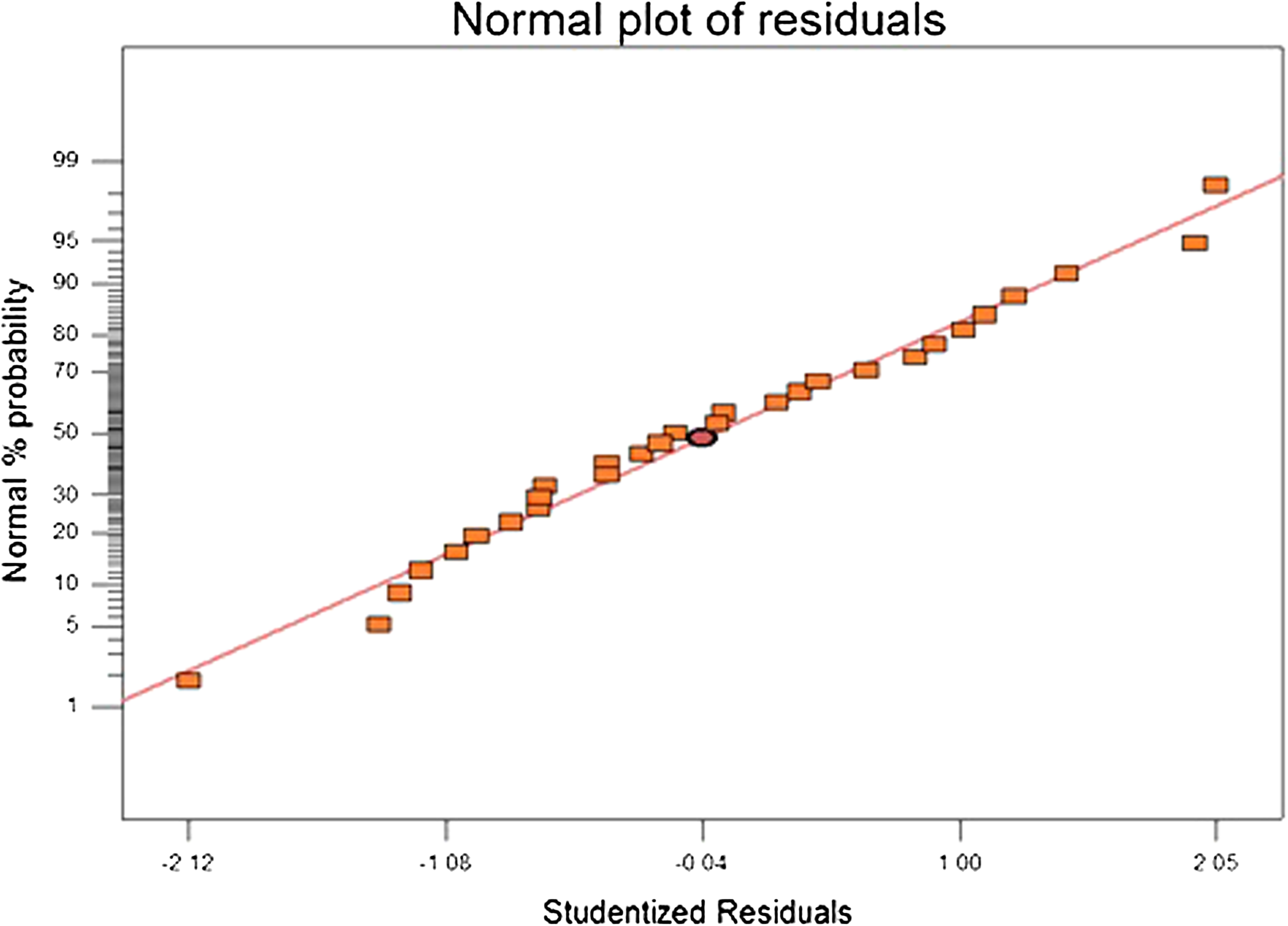

Supplement: Supplementary file 5 — Authors’ original file for figure 5 [file 40201_2012_5149_MOESM5_ESM.tif]

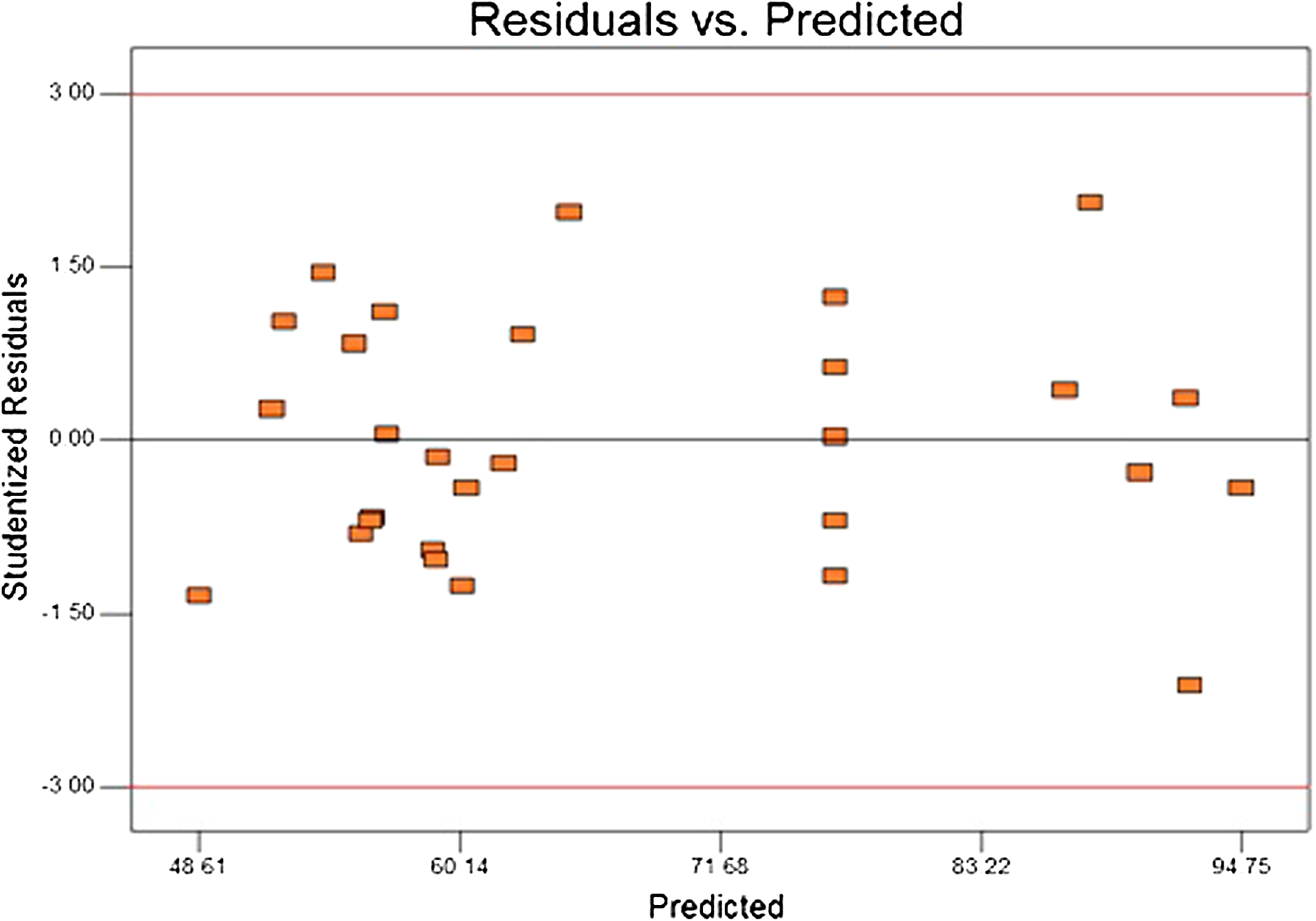

Supplement: Supplementary file 6 — Authors’ original file for figure 6 [file 40201_2012_5149_MOESM6_ESM.tif]

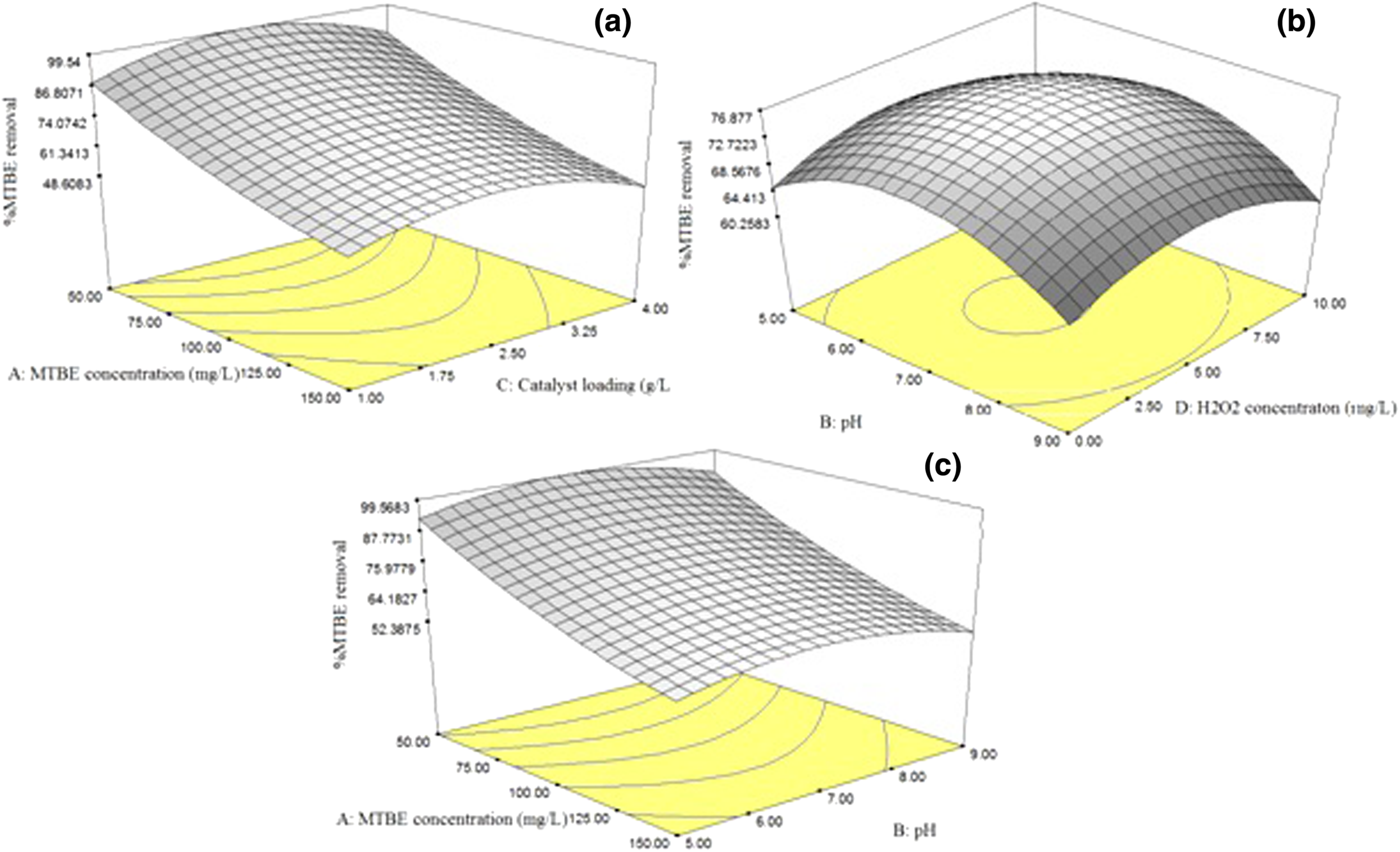

Supplement: Supplementary file 7 — Authors’ original file for figure 7 [file 40201_2012_5149_MOESM7_ESM.tif]

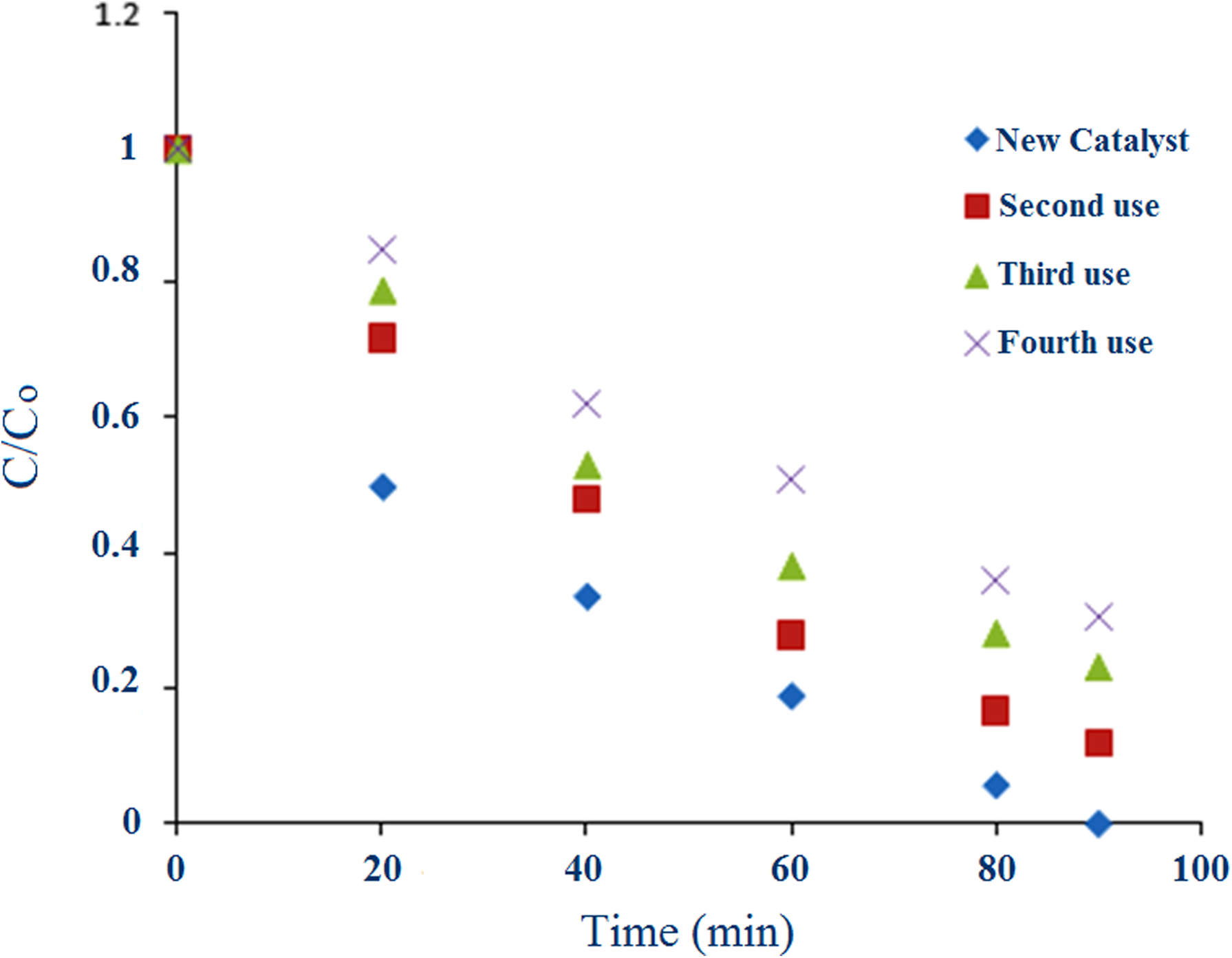

Supplement: Supplementary file 8 — Authors’ original file for figure 8 [file 40201_2012_5149_MOESM8_ESM.tiff]
